# Highly suppressed solar absorption in a daytime radiative cooler designed by genetic algorithm

**DOI:** 10.1515/nanoph-2021-0436

**Published:** 2021-11-09

**Authors:** Sunae So, Younghwan Yang, Soomin Son, Dasol Lee, Dongwoo Chae, Heon Lee, Junsuk Rho

**Affiliations:** Department of Mechanical Engineering, Pohang University of Science and Technology (POSTECH), Pohang 37673, Republic of Korea; Department of Materials Science and Engineering, Korea University, Seoul 02841, Republic of Korea; Department of Biomedical Engineering, Yonsei University, Wonju 26493, Republic of Korea; Department of Chemical Engineering, Pohang University of Science and Technology (POSTECH), Pohang 37673, Republic of Korea; POSCO-POSTECH-RIST Convergence Research Center for Flat Optics and Metaphotonics, Pohang 37673, Republic of Korea

**Keywords:** computational optimization, multilayer structures, radiative cooling, selective emitters

## Abstract

Here, we report a selective multilayer emitter for eco-friendly daytime passive radiative cooling. The types of materials and thickness of up to 10 layers of the multilayer structure are optimized by a genetic algorithm. The passive radiative cooler is designed to mainly target low solar absorption, which allows sub-ambient cooling under direct sunlight. We used a custom objective function in the solar region to achieve high-performance daytime radiative cooling to minimize solar absorption. The designed structure minimizes solar absorption with an average absorptivity of 5.0% in the solar region (0.3–2.5 μm) while strongly emitting thermal radiation with an average emissivity of 86.0% in the atmospheric transparency window (8–13 μm). The designed and fabricated structure achieves daytime net cooling flux of 84.8 W m^−2^ and 70.6 W m^−2^, respectively, under the direct AM 1.5 solar irradiation (SI) (total heat flux of 892 W m^−2^ in the 0.3–2.5 μm wavelength region). Finally, we experimentally demonstrate a passive radiative cooling of the fabricated selective emitter through a 72-hour day-night cycle, showing an average and maximum temperature reduction of 3.1 °C and 6.0 °C, respectively. Our approach provides additional degrees of freedom by designing both materials and thickness and thereby is expected to allow high-performance daytime radiative cooling.

## 1 Introduction

Passive radiative cooling strategy cools the temperature of structures without external active devices and thereby any energy consumption [1–4]. It dissipates heat away into exterior space of around ∼3 K temperature through the atmospheric transparency window (8–13 μm). The radiative heat transfer attributes to the coincidence of the wavelength region between the electromagnetic transparent window and the blackbody radiation at typical ambient temperature (∼300 K). Accordingly, extensive studies have focused on cooling down the temperature by maximizing light emission in the transparency window for nighttime radiative cooling [1, 5], [6], [7]. However, with the presence of SI, it is also crucial for daytime radiative cooling to suppress additional undesired heat absorbed by the Sun. Therefore, for high-performance daytime radiative cooling, devices should strongly reflect sunlight in the solar spectral region (0.3–2.5 μm), while strongly emit thermal radiation in the atmospheric transparency region (8–13 μm) at the same time. So far, several radiative coolers are elaborately designed for daytime radiative cooling with one-dimensional (1D) photonic film structures [3, 8], [9], [10], [11], [12], [13], microstructures [14–17], nanostructures [18–22], and random particles [23–25].

The ideal case of the daytime radiative cooler is the selective emitter, the emissivity of which is one in the transparency window in (8–13 μm), and zero elsewhere [18, 20, 26]. However, designing such an ideal selective emitter is quite formidable, so many efforts have been made to realize structures that minimize absorption in the solar spectral region while maximizing emission in the transparency window [18, 27, 28]. Indeed, Rephaeli et al. have reported that only 10 percent absorption in the solar spectral region resulted in no cooling effect even with the selective emitter in the transparency region [18]. Considering that even a tiny amount of solar power absorbed by a device can substantially degrade the cooling performance, solar absorption should be considered a more critical factor when designing a high-performance daytime radiative cooling device.

In this study, we report a high-performance daytime radiative cooler that is designed to minimize solar absorption power. To optimize multilayer emitter for high-performance daytime radiative cooling, we develop a weighted objective function [29, 30] that takes into account the weighted sum of solar absorption and other cooling power factors. Using a genetic algorithm (GA) [31], the types of materials, as well as thicknesses of up to 10 layers of photonic films, were designed for high-performance daytime radiative cooling. The optimization approach is expected to increase additional degrees of freedom. Among the four material candidates of silicon dioxide (SiO_2_) [32], silicon nitride (Si_3_N_4_) [32], magnesium fluoride (MgF_2_) [33], and hafnium dioxide (HfO_2_) [33], proper materials were recommended, and thicknesses were optimized for desired optical functionalities. Finally, we experimentally demonstrate a selective radiative-cooled emitter of a design designed with a 72 hour day-night cycle in outdoor conditions.

## 2 Results and discussion

### 2.1 Design results

The net cooling flux (*Q*_net_) of a structure with the unit area can be driven from the energy conservation law [3, 34]. Under the SI and ambient temperature of *T*_amb_, *Q*_net_ is given by:

(1) $$\begin{array}{ll} Q_{\text{net}}\left(T,T_{\text{amb}}\right) & =Q_{\text{rad}}\left(T\right)-Q_{\text{atm}}\left(T_{\text{amb}}\right)\\ & \;-Q_{\text{Sun}}-Q_{\text{nonrad}}\left(T,T_{\text{amb}}\right), \end{array}$$

where,

(2) $$Q_{\text{rad}}\left(T\right)=\int \overset{\infty }{\underset{0}{\int }}I_{\text{BB}}\left(T,\lambda \right)\epsilon \left(\lambda ,\theta \right)d\lambda \;\cos\theta \;d\Omega$$

is the flux radiated out by the sample structure.

(3) $$\begin{array}{ll} Q_{\text{atm}}\left(T_{\text{atm}}\right) & =\int \overset{\infty }{\underset{0}{\int }}I_{\text{BB}}\left(T_{\text{atm}},\lambda \right)\epsilon \left(\lambda ,\theta \right)\epsilon_{\text{atm}}\\ & \;\times \left(\lambda ,\theta \right)d\lambda d\;\cos\theta \;d\Omega \end{array}$$

is the flux absorbed by atmospheric heat exchange outside the atmospheric window.

(4) $$Q_{\text{Sun}}=\overset{\infty }{\underset{0}{\int }}I_{\text{AM}1.5}\left(\lambda \right)\epsilon \left(\lambda ,\theta \right)d\lambda$$

is the absorbed heat flux due to incident SI.

(5) $$Q_{\text{nonrad}}\left(T,T_{\text{amb}}\right)=h_{c}\left(T_{\text{atm}}-T\right)$$

is the flux lost due to nonradiative heat transfer of convection and conduction. Here, *λ* is the wavelength, *θ* is the polar angle, $\int d\Omega =2\pi \int_{0}^{\pi /2}\;\sin\theta d\theta$ is angular integral over a hemisphere, and *I*_AM1.5_(*λ*) is a solar illumination of the AM1.5 spectrum. *I*_BB_ is the spectral black-body radiation at temperature *T* of $I_{\text{BB}}\left(T,\lambda \right)=\frac{2hc^{2}}{\lambda^{5}}\left(\frac{1}{e^{hc/\left(\lambda k_{\text{B}}T\right)}-1}\right)$ , where *h* is Planck’s constant, *c* is the speed of light in a vacuum, and *k*_B_ is the Boltzmann constant. *ϵ*(*λ*, *θ*) is the spectral angular emissivity of the sample, and *ϵ*_atm(*λ*,*θ*)_ = 1 − *t*(*λ*)^1/cos(*θ*)^ is the spectral angular emissivity of the atmosphere, where *t*(*λ*) is the atmospheric transmittance in the zenith direction [34]. We obtained the emissivity from Kirchhoff’s law of thermal radiation [35], where the emissivity is equal to the absorptivity in a thermodynamic equilibrium state.

**Figure 1:**
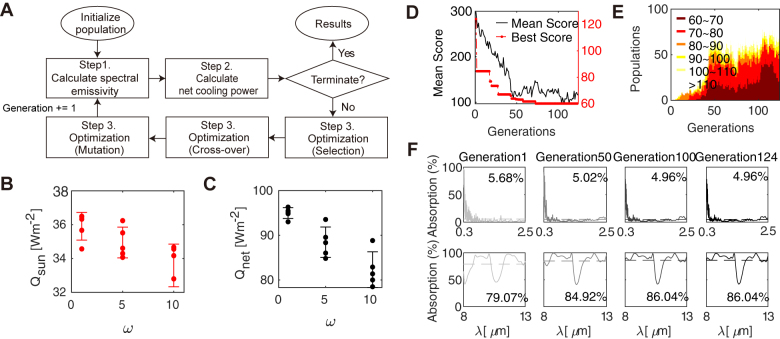
GA optimization results for up to 10 layer selective emitter. (A) Flow chart of GA optimization for the radiative cooling emitter. Weight analysis results in objective function l. Five different optimizations were performed using a GA for three weights *ω* = 1, 5, and 10, and (B) *Q*_Sun_ and (C) *Q*_net_ were calculated for the final optimized structure. (D) The progress of the optimization over the generation is shown with the averaged (black solid line) and the best (red dotted line) score of the objective function. (E) Distribution of objective function scores per generation. From a total of 100 populations, the number of individuals that fall within the range of scores for the objective function is counted. The legend indicates the corresponding score range for the objective function. (F) Absorption spectra in the solar region (top) and in the transparency window (bottom) region of the best solution in each generation.

Among the thermal flux terms in Eqs. (1)–(5), *Q*_Sun_ is of particular importance because even a small amount of solar power absorbed by a device can significantly degrade the cooling performance. In addition, it is relatively less dependent on environmental conditions than other flux terms, as it is only affected by SI and emissivity spectrum from the UV to near-IR region. Therefore, we aimed to design a selective emitter that minimizes *Q*_Sun_. For this, we developed an objective function (*l*) that takes into account the weighted sum of *Q*_Sun_ and other cooling power factors as given by

(6) $$l=-\left(Q_{\text{rad}}-Q_{\text{atm}}\left(T_{\text{atm}}\right)-\omega \times Q_{\text{Sun}}\right),$$

where *ω* is the weight, and *T*_atm_ = 300 K is used in optimization. The optimization was performed to minimize the objective function in Eq. (6). To optimize the design of up to 10 layer multilayer emitter, we used an evolutionary optimization algorithm of a GA that is inspired by natural evolution theory. As the optimization method progressed, the solution was improved through iterative operations of selection, mutation, and crossover (Figure 1A). In order to expand the design space for optimization, we optimized not only the structural parameter of thicknesses but also the material type of each layer for up to 10 layer multilayer emitter. For multilayer structures, many dielectric materials can be fabricated using thin film deposition. Among the many possible materials in nature, the following four materials were selected: SiO_2_, Si_3_N_4_, MgF_2_, and HfO_2_ for their excellent chemical/thermal resistance and durability. The chosen four material candidates are then indexed for material optimization (Table 1). Through optimization, appropriate materials are recommended, and thicknesses are designed for the desired optical function.

**Table 1:** Indexed materials.

0	1	2	3	4
None	SiO_2_	Si_3_N_4_	MgF_2_	HfO_2_

The multilayer structure to be designed consists of up to 10 layers of 1D photonic crystal on top of silver reflecting layer. We also added a layer of Polydimethylsiloxane (PDMS) on top, which is known to have a high transmittance in the solar region and emissivity at the atmospheric window. The middle 10 layer of 1D photonic crystals and a bottom silver layer reduce reflection in the solar region. The top PDMS is responsible for high emissivity in the atmospheric window. Overall, GA was performed to optimize the total 21 variables of the thickness of the top PDMS layer, and types of materials and thickness of each layer in the middle 10 layers (Table 2). A rigorous coupled-wave analysis (RCWA) method was used to obtain absorption spectra.

**Table 2:** Variables to be optimized.

	1	2	…	11	12	…	21
Variables	*t*_PDMS_	*t*_Layer 1_	…	*t*_Layer 10_	*m*_Layer 1_	…	*m*_Layer 10_
Variable range	(0–10 μm)	(0–1 μm)	…	(0–1 μm)	0–4	…	0–4

To determine appropriate weights in *l*, we performed five optimizations each for three different weights *ω* = 1, 5, and 10, and the optimized *Q*_Sun_ and *Q*_net_ are shown in Figure 1B and C. Not surprisingly, it can be seen that the solar absorption decreases with increasing weights due to the weight of the objective function, but the total optimized net cooling flux also decreases. These results suggest that weights can be given to important factors in the optimization process through a ‘weighted objective function’. Therefore, we chose the weight *ω* = 5 to minimize solar absorption without decreasing net cooling flux too much. For *ω* = 5, the optimal thicknesses and materials of the multilayer were designed with 124 generations of a total of 11,696 calculations. Figure 1D and E summarize the results of GA with the objective function values, and the population distribution per generation, respectively. As generation progresses, the number of individuals with low scores for the objective function in the entire population gradually increases, indicating evolution (Figure 1E). The progress of the optimization process, which plots the best solution in each generation, shows that the absorption in the solar region is reduced from 5.7% to 5.0%, and the emissivity in the atmospheric window is increased from 79.0% to 86.0% (Figure 1F). In the solar region, the 1D photonic crystals of alternating high- and low-index materials allow additional reflection compared to that of single silver films (5.2%).

### 2.2 Experimental results

**Figure 2:**
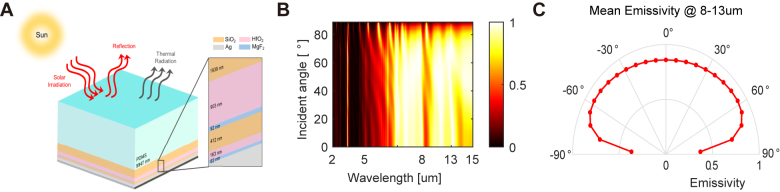
Simulation results of the optimized structure. (A) Schematics of the optimized design structure. (B) Emissivity/absorptivity spectra of the designed structures for different incident angles. (C) Mean angular emissivity of the designed structure.

The designed multilayer structure for optimal daytime radiative cooling is composed of total of eight layers including top PDMS and bottom silver layers (Figure 2A). The optical absorptivity/emissivity of the designed structure was evaluated by RCWA simulation. The calculated absorption/emissivity spectrum showed strong light emission in the atmospheric window for all angles of incident light (Figure 2B). The average angular emissivity in the atmospheric window was also higher than 80% for the incident angle from 0° to 70° (Figure 2C). Such a broad-angle emissivity is desirable for daytime radiative cooling, especially when nonradiative heat transfer is non-negligible, and the sample temperature is higher than the ambient temperature [36].

**Figure 3:**
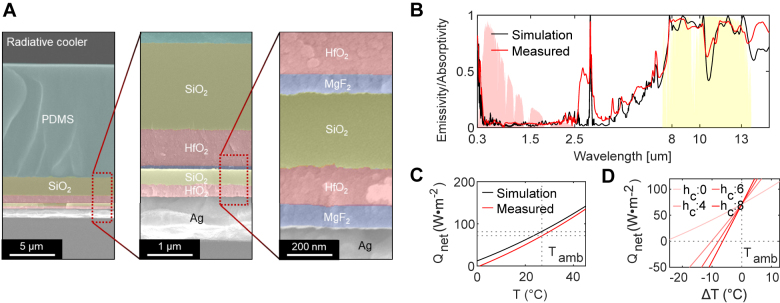
Experimental results. (A) SEM image of the fabricated sample. (B) Absorptivity/emissivity spectra of the designed structure (black line and blue dot: calculated by RCWA and FEM simulations, respectively) and the fabricated structure (red line) at a zero-incident angle. (C) Calculated net cooling flux and (D) calculated cooling temperature based on the emissivity/absorptivity spectra with various heat transfer coefficients of *h*_c_ = 0, 4, 6, 8 W m^−2^ K^−1^.

The optimized multilayer structure was then fabricated for the experimental demonstration. The designed structure composed of MgF_2_, HfO_2_, SiO_2_, and PDMS layers were deposited on an Ag-coated silicon wafer (Figure 3A). The first MgF_2_, HfO_2_, and SiO_2_ layers were deposited with a thickness of 62, 163, and 412 nm, respectively. Again, the second MgF_2_, HfO_2_, and SiO_2_ layers were fabricated with a thickness of 92, 903, and 1939 nm, respectively. Within the above process, Ag, MgF_2_, and HfO_2_ layers were deposited with an electron beam evaporator and SiO_2_ layers were fabricated by plasma-enhanced chemical vapor deposition. Lastly, the top PDMS layer was spin-coated using 4500 rpm on the deposited multilayer and then baked at 60 °C for 2 h.

The emissivity/absorptivity of the fabricated sample was measured by Fourier-transform infrared spectroscopy and compared with a simulation result (Figure 3B). We additionally performed numerical simulation based on finite element method (FEM) using the commercially available FEM solver, COMSOL Multiphysics, in addition to RCWA calculation. Both simulations and experimental results show wavelength-selective properties well, where light absorption is highly suppressed in the solar region and the light is strongly emitted in the atmospheric window. The optical spectra show overall good agreement except for the effect of the carbon dioxide and water vapor in the 3–4 μm region. The average emissivity/absorptivity of the simulation result is 5.0% in the solar region and is 86.0% in the atmospheric transparency window. The average emissivity/absorptivity of the experimental result is 6.7% in the solar region and is 88.0% in the atmospheric transparency window. Using the calculated and measured optical properties, the net cooling flux was calculated from Eq. (1). To calculate cooling flux, we use the AM1.5G SI spectrum with a total heat flux of 892 W m^−2^ in the 0.3–2.5 μm wavelength region. The net cooling flux for an ideal case (*P*_nonrad_ = 0) at the ambient temperature of *T*_amb_ = 300 K for simulation and experimental results were 84.8 W m^−2^ and 70.6 W m^−2^, respectively (Figure 3C). However, additional power losses occur in practice due to the nonradiative heat transfer of convection and conduction. The four heat transfer coefficients *h*_c_ = 0, 4, 6, 8 W m^−2^ K^−1^ are used to calculate the net cooling flux representing various environmental conditions (Figure 3D). In an ideal case, where the heat transfer coefficient is zero, the fabricated selective emitter can cool the temperature of the sample up to 25 °C by reaching a thermal equilibrium state. The cooling temperature decreases to 10.2 °C, 7.9 °C, and 6.5 °C for heat transfer coefficients of *h*_c_ = 4 W m^−2^ K^−1^, 6 W m^−2^ K^−1^, and 8 W m^−2^ K^−1^, respectively.

**Figure 4:**
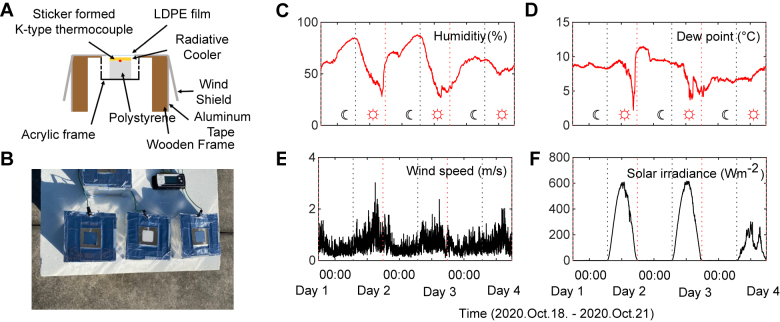
Temperature measurement setup and the measured environment. (A) A schematic and (B) a photograph of the custom outdoor temperature measurement system. Measured (C) humidity, (D) dew point, (E) wind speed, and (F) SI during the temperature measurement period (2020.Oct.18–2020.Oct.21). The sunrise and sunset time is indicated by a red and black dashed line, respectively.

Finally, sub-ambient radiative cooling through a 72 hour day-night cycle was experimentally demonstrated using the selective emitter (Figure 4). The custom outdoor temperature measurement system was constructed with a wooden frame chamber and acrylic plates (Figure 4A). The wooden frame was wrapped with aluminum tape to reflect sunlight and, hence, to avoid additional unnecessary solar heating. Additionally, there is a windshield to minimize the influence of the wind, and holes are drilled on the side of the acrylic frame to reduce the temperature inside the acrylic, which increases excessively during the daytime. Polystyrene was used to support the sample and acted as a thermal insulator. The K-type thermocouple was used to measure the temperature of the sample. The additional thermocouple was installed inside the same chamber to measure the inner ambient temperature for comparison. The custom measurement system measured the temperature of the sample and inner ambient at 30 s intervals. A commercial white paint sample and a bare silver film were also prepared to compare the cooling performance of the radiative cooler (Figure 4B). In addition, the environmental conditions, such as SI, dew point, humidity, and wind speed, were measured by the commercial weather station.

Using the outdoor rooftop temperature measurement setup, temperatures of the radiative cooler were measured for 72 h day-night cycle in Seoul (37° 58′N, 127° 02′E), Republic of Korea (2020.Oct.18–2020.Oct.21). As the cooling effect of the radiative cooler is highly affected by environmental conditions, the weather conditions of the humidity, dew point, wind speed, and SI were also measured during the same period (Figure 4C–F). The average sunrise time and sunset time during the 72 h measurement period in Seoul, Republic of Korea, was 6:44 AM and 5:49 PM, respectively. During the measurement period, the relative humidity was very high at night showing the average and maximum humidity of 61.0%, and 87.9%, respectively (Figure 4C). In addition, the average dew point over 72 h was 7.9 °C, which represents a significant amount of moisture in the air (Figure 4D). These could lead to the substantial degradation of the radiative cooling performance by lowering the atmospheric transmittance [8, 37], [38], [39].

**Figure 5:**
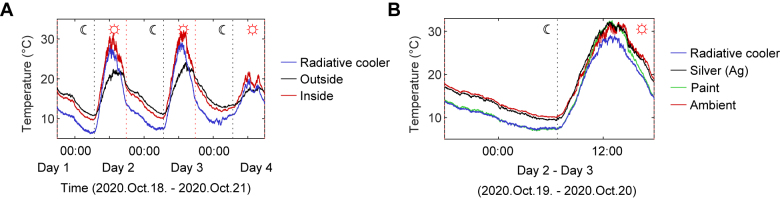
Measured temperatures. (A) Measured temperatures of the radiative cooler (blue), inner ambient (red), and outside (black) for a 72 hour day-night cycle in Seoul, Republic of Korea. (B) Measured temperatures of three samples of the radiative cooler (blue), commercial white paint (green), and bare silver film (black) with an inner ambient temperature (red).

Figure 5 shows the measured temperature over 72 h day-night cycle (2020.Oct.18–2020.Oct.21). The measurement chamber was thermally insulated with low-density polyethylene film, so the inner ambient temperature was higher than the outside during the daytime and lower at night (Figure 5A). During the entire measurement time, the temperature of the radiative cooler was kept below the inner ambient temperature, indicating that the radiative cooler performed well. During the 72 h day-night measurement period, an average and maximum temperature reduction of 3.1 °C and 6.0 °C, respectively, was achieved compared to inner ambient. It should be noted that the radiative cooler still showed a substantial cooling performance during the nighttime with an average cooling temperature of 3.5 °C despite the unfavorable weather conditions of high relative humidity and dew point. During the daytime on Day 4, the weather was foggy, so the amounts of SI were low (Figure 4F). Accordingly, the cooling performance degraded due to the low outside temperature. Overall, the radiative cooler showed a cooling capability for various weather conditions over a full-time 72 hour day-night cycle. The average and maximum cooling performance for each day and night time over 72 h are summarized in Table 3 with featured weather conditions.

**Table 3:** Cooling performance of the radiative cooler over 72 h.

	Day 1	Day 2		Day 3		Day 4	72-h
	Night	Day	Night	Day	Night	Day	
Max Δ*T* (°C)	4.3	5.2	4.4	6.0	5.2	3.0	6.0
Avg Δ*T* (°C)	3.7	3.0	3.6	3.1	3.3	2.0	3.1
Max humidity (%)	85.0	84.4	87.9	86.6	67.1	64.1	87.9
Avg humidity (%)	70.7	54.1	75.5	49.4	57.1	56.0	61.0
Max dew point (°C)	9.1	10.7	11.5	9.2	7.1	8.6	11.5
Avg dew point (°C)	8.6	8.2	10.0	7.1	6.3	7.3	7.9
Max SI (W m^−2^)	–	609	–	616	–	301	616
Avg SI (W m^−2^)	–	339	–	345	–	119	123

To further investigate the cooling performance of the radiative cooler, temperatures of a bare Ag film and a commercial white paint (shown in Figure 4B) were also measured to serve as comparison samples under the same weather condition (Figure 5B). During the 72 hour measurement period, the average temperature of the cooler was 2.9 °C, 0.7 °C lower than that of a silver film and a paint, respectively. As the cooler is specifically designed to minimize *Q*_Sun_, the temperature difference with two comparison samples during the daytime was larger, showing 3.2 °C, and 1.6 °C, respectively. Due to the selective emitting properties, the radiative cooler outperformed a single reflector of bare silver film and a broadband emitter of commercial white paint.

Finally, we summarize the previous studies for the daytime radiative cooler for comparison (Table 4). Various structured devices with composites, polymers, micro/nanoparticles, and multilayered films have been demonstrated for the state-of-the-art radiative coolers. Using a GA, we demonstrated highly suppressed solar absorption and high emission in the transparency window in the multilayered structures. Under direct SI, the designed and fabricated structure achieves high-performance daytime radiative cooling with a net cooling flux of 84.8 W m^−2^ and 70.6 W m^−2^, respectively. 

**Table 4:** Comparison of the performances with other radiative coolers.

Ref.	Building block	Average solar absorptivity	Average emissivity (8–13 μm)	Net cooling power (W m^−2^)
[40]	Polymer composites	5.0%^b^	96%^b^	90.8^b^
[17]	Porous composites	14.0%^b^	96%^b^	65.6^b^
[41]	Porous polymer	7.0%^b^	94%^b^	102^b^
[11]	Nanoparticles	3.5%^b^	91%^b^	98.9^b^
[23]	Micrspheres	4.0%^b^	93%^b^	93.0^b^
[3]	Multilayer	3.0%^b^	68%^b^	40.1^b^
[42]	Multilayer	3.0%^a^	96%^a^	83.0^a^
[9]	Multilayer	5.6%^a^, 5.2%^b^	91%^a^, 87%^b^	73.8^a^, 66.4^b^
This work	Multilayer	5.0%^a^, 6.5%^b^	86%^a^, 88%^b^	84.8^a^, 70.6^b^

^a^Simulation results.

^b^Experimental results.

## 3 Conclusions

In this study, a selective multilayer emitter was designed by a GA for daytime radiative cooling. The passive radiative cooler was specifically designed for low solar absorption, allowing sub-ambient radiative cooling. Using a GA, types of materials and thickness of up to 10 layers of the multilayer structure were optimized. The designed structure was then fabricated for the experimental demonstration, and it allowed the net cooling flux of 70.6 W m^−2^ under direct sunlight. Through a custom rooftop temperature measurement system, the outdoor temperature of the radiative cooler was measured, showing an average and maximum temperature reduction of 3.1 °C and 6.0 °C, respectively, compared to the inner ambient temperature. The sub-ambient radiative cooling was achieved even with the unfavorable weather conditions with high relative humidity and dew point during the nighttime. We believe the cooling performance could be improved with better weather conditions. Our design method is expected to allow high-performance daytime radiative cooling by designing both material and thickness to provide additional degrees of freedom.
